# The development and nutritional quality of *Lyophyllum decastes* affected by monochromatic or mixed light provided by light-emitting diode

**DOI:** 10.3389/fnut.2024.1404138

**Published:** 2024-05-27

**Authors:** Xiaoli Chen, Yihan Liu, Wenzhong Guo, Mingfei Wang, Jiuxiao Zhao, Xin Zhang, Wengang Zheng

**Affiliations:** ^1^Intelligent Equipment Research Center, Beijing Academy of Agriculture and Forestry Sciences, Beijing, China; ^2^College of Horticultural and Landscape Architecture, Tianjin Agricultural University, Tianjin, China

**Keywords:** edible fungi, *Lyophyllum decastes*, nutritional quality, light quality, extracellular enzymes, photoreceptor

## Abstract

Edible fungi has certain photo-sensitivity during the mushroom emergence stage, but there has been few relevant studies on the responses of *Lyophyllum decastes* to different light quality. *L. decastes* were planted in growth chambers with different light qualities that were, respectively, white light (CK), monochromatic red light (R), monochromatic blue light (B), mixed red and blue light (RB), and the mixture of far-red and blue light (FrB). The photo-sensitivity of *L. decastes* was investigated by analyzing the growth characteristics, nutritional quality, extracellular enzymes as well as the light photoreceptor genes in mushroom exposed to different light treatments. The results showed that R led to mycelium degeneration, fungal skin inactivation and failure of primordial formation in *L. decastes*. The stipe length, stipe diameter, pileus diameter and the weight of fruiting bodies exposed to RB significantly increased by 8.0, 28.7, 18.3, and 58.2% respectively, compared to the control (*p* < 0.05). B significantly decreased the stipe length and the weight of fruiting body, with a decrease of 8.5 and 20.2% respectively, compared to the control (*p* < 0.05). Increased color indicators and deepened simulated color were detected in *L. decastes* pileus treated with B and FrB in relative to the control. Meanwhile, the expression levels of blue photoreceptor genes such as *WC-1*, *WC-2* and *Cry-DASH* were significantly up-regulated in mushroom exposed to B and FrB (*p* < 0.05). Additionally, the contents of crude protein and crude polysaccharide in pileus treated with RB were, respectively, increased by 26.5 and 9.4% compared to the control, while those in stipes increased by 5.3 and 58.8%, respectively. Meanwhile, the activities of extracellular enzyme such as cellulase, hemicellulase, laccase, manganese peroxidase, lignin peroxidase and amylase were significant up-regulated in mushroom subjected to RB (*p* < 0.05), which may promote the degradation of the culture materials. On the whole, the largest volume and weight as well as the highest contents of nutrients were all detected in *L. decastes* treated with RB. The study provided a theoretical basis for the regulation of light environment in the industrial production of high quality *L. decastes*.

## 1 Introduction

*Lyophyllum decastes* (*L. decastes*), also known as fried chicken mushroom, belongs to the order Agaricales, family Tricholomataceae, and genus Lyophyllum. It is named for its resemblance to the medicinal plant pilose antler (1). *L. decastes* is a large fungus widely distributed in temperate regions of the northern hemisphere, which can be used for both food and medicine. The substances such as protein, vitamins, dietary fiber, minerals, amino acids and trace elements in the mycelium and fruiting body have high nutritional value, while the crude polysaccharides have medicinal functions such as antioxidant, anti-tumor, blood pressure lowering, cholesterol lowering, and immune function improving (2, 3). Nowadays, the market prospect of *L. decastes* is promising due to its rich nutritional composition, good medicinal properties and rich flavor.

The growth and development process of edible fungi can be divided into the nutritional growth stage and the reproductive growth stage, and the transition between these two stages is mainly achieved through changes in environmental factors. Among them, light is one of the important environmental factors affecting the growth and development of edible fungi. Some studies have shown that certain mushrooms such as *Agaricus bitorquis*, *Agaricus bisporus*, and underground *Poria cocos* can produce fruiting bodies in total darkness. However, light is indispensable during the reproductive growth stage of most edible mushrooms. Meanwhile, studies have reported that the light requirements of edible fungi are related to the variety and growth stage (4–6). Previous studies have reported that light quality can affect the mycelium activity, as well as the color, size and weight of the fruiting bodies, and edible fungi exhibited different fruiting body morphologies under different light environments. Wu et al. (7) showed that red and yellow light promoted the mycelial growth of *Pleurotus eryngii* in solid culture compared with the white light. Dong et al. (8) showed that pink light increased the dry matter content of *Cordyceps militaris* fruiting body compared with the blue light. Yu et al. (9) investigated the effects of light quality on the growth of mycelium and fruiting bodies of *Volvariella volvacea*, and found that blue-green light was beneficial for the growth of mycelium, and the number and yield of fruiting bodies were also the highest under blue-green light. Song et al. (10) found that the length and diameter of the *Hypsizygus marmoreus* stipe were the largest in the dark, while the pileus diameter was the largest under blue light and the combination of red, green and blue light. Light quality not only affected the growth of mycelium and fruiting body, but also affected the synthesis and accumulation of nutrient substance in edible fungi. Jang and Lee (11) showed that the yield and ergot content of *Pleurotus ostreatus* were higher under mixed blue and white light in relative to the white light. Wu et al. (7) showed that the exopolysaccharides (EPS) production of *Pleurotus eryngii* was highest under blue light conditions. In addition, studies have shown that light exposure time also had impacts on the development of edible fungi. Li et al. (12) reported that 6 h of light exposure was beneficial for the appearance and total yield (per bottle) of fruiting bodies, while 18 h of light exposure was beneficial for the weight of individual fruiting body.

The nutrients required for the growth and development of edible fungi are mainly provided by cultivation materials. Edible fungi decompose macromolecular substances into micromolecular substances under the catalytic action of extracellular enzymes. The small molecule substances which are easily absorbed and transformed by the mycelium and fruiting bodies, can provide nutrients for the hypha growth, primordial formation, and fruiting body growth. Therefore, extracellular enzymes play a crucial role in edible fungi growth and development. The main extracellular enzymes during the growth of edible fungi include cellulase system, hemicellulase system, lignin degrading enzyme system, and amylase system (13–15). Cellulases include endo-1,4-β-D-glucanohydrolase (E.C.3.2.1.4), exo-1,4-β-D-glucanase (E.C.3. 2.1.91) and β-1,4- glucosidase (E.C.3.2.1.21), etc. Hemicellulases include endo-1,4-β-xylanase (E.C.3.2.1.8) and exo-1,4-β-xylosidase (E.C.3.2.1.37), etc. Lignin degrading enzymes include Lignin peroxidase (E.C.1.11.1.14), Manganese peroxidase (E.C.1.11.1.13) and Laccase (E.C.1.10.3.2). Amylase includes α-Amylase (E.C.3.2.1.1), β-Amylase (E.C.3.2.1.2), Glucoamylase (E.C.3.2.1.3.) and Isoamylase (E.C.3.2.1.68) (16). Study has shown that green light can increase the activities of total cellulase, endo-1,4-β-D-glucanohydrolase, and xylanase in *Pleurotus ostreatus*, but reduce the activity of laccase (17). The extracellular enzyme activity of edible fungi varies in different environments, which reflects the growth rate of mycelium and its ability to absorb small molecule nutrients, thereby affecting the yield and nutrient quality of edible fungi. Therefore, it is of great practical significance to study the response of extracellular enzymes in edible fungi to different light qualities.

So far, studies on the effects of light on the growth and development of edible fungi mainly focus on *Auricularia auricula*, *Flammulina velutipes*, *Ganoderma lucidum*, and *Lentinula edodes*, etc. On the contrary, there have been few studies on the effects of artificial lights on the characteristic mushroom *L. decastes*. This study not only analyzed the impacts of light quality on *L. decastes* from the perspective of phenotype, but also further explored the mechanism of light’s influence on the mushroom at the molecular level. Meanwhile, light emitting diodes (LED) control system with all-round adjustable light formula has been applied in the experiment, ensuring the stability and accuracy of the lighting environment. The results are expected to provide a theoretical basis for the light regulation in the industrial production of high-quality *L. decastes*.

## 2 Materials and methods

### 2.1 Experimental design

This experiment was conducted in a growth chamber of BAAFS, Beijing, China, using an LED system that can set any light formula. The *L. decastes* mushroom-sticks were treated with different light qualities from the day when the mycelium was full. The growth cycle of the mushrooms were divided into four stages that were hyphal stage [needle shaped primordium, undifferentiated fruiting body, 0–14 days after treatment (DAT)], bud stage (hemispherical pileus, 15–25 DAT), coralline stage (oblate hemispherical pileus, 26–31 DAT), and mature stage (flat pileus, diameter ≥ 35 mm, 32–37 DAT) (18).

Five treatments were set up in the experiment, namely white light (CK), pure blue light (B), pure red light (R), red and blue light (RB), and far-red and blue light (FrB), with a total light intensity of 15 μmol·m^−2^·s^−1^ for each treatment. The wavelength peak of blue light, red light and far-red light were, respectively, 450 nm, 660 nm and 735 nm. The light intensity and spectrum were all measured approximately 10 cm below the light source using a spectrometer (LI-180, LI-COR, USA). The light/dark (L/D) period at hyphal stage, bud stage, coralline stage and mature stage were, respectively, 12 h/12 h, 12 h/12 h, 22.5 h/1.5 h and 21 h/3 h (Supplementary Figure S1 and Table 1). The temperature and the CO_2_ concentration in the growth chamber was, respectively, 17 ± 1°C and 1,550 μmol·mol^−1^, and the relative humidity of air was, respectively, (80 ± 1)%, (80 ± 1)%, (90 ± 1)% and (90 ± 1)% at hyphal stage, bud stage, coralline stage and mature stage. Purified water was sprayed three time per day with a spray bottle during the growth period.

**Table 1:** Irradiation modes of LED light in different treatments.

Treatment		Light supply mode	Light intensity (μmol·m^−2^·s^−1^)			
			Blue light	Red light	White light	Far-red light
Hyphal stage (0–14 DAT) &Bud stage (15–25 DAT) L(12 h) / D(12 h)	B	pure blue light	15	0	0	0
	R	pure red light	0	15	0	0
	CK	white light	0	0	15	0
	RB	red and blue light	7.5	7.5	0	0
	FrB	far-red and blue light	7.5	0	0	7.5
Coralline stage (26–31 DAT) L(22.5 h) / D(1.5 h)	B	pure blue light	15	0	0	0
	R	pure red light	0	15	0	0
	CK	white light	0	0	15	0
	RB	red and blue light	7.5	7.5	0	0
	FrB	far-red and blue light	7.5	0	0	7.5
Mature stage (32–37 DAT) L(21 h) / D(3 h)	B	pure blue light	15	0	0	0
	R	pure red light	0	15	0	0
	CK	white light	0	0	15	0
	RB	red and blue light	7.5	7.5	0	0
	FrB	far-red and blue light	7.5	0	0	7.5

### 2.2 Sampling and phenotypic measurement

The length and diameter of mushroom stipe, the pileus diameter as well as the mushroom-sticks weight of *L. decastes* were dynamically measured at 25, 28, 31, 34 and 37 DAT. The weight of fruiting body was measured at harvest (37 DAT). Eight fruiting bodies randomly taken from per treatment was regarded as a repetition, and there were three repetitions in each treatment.

The color parameters and the spectral reflectance ranging 400 ~ 700 nm of the fruiting body were measured by spectrophotometer (Shenzhen San’enshi Technology Co., Ltd., Guangzhou, China) at harvest. The color tone was obtained using various color space parameters (L*, a* and b*). L* represents the gloss brightness, with a value range of [0,100]. The larger L*, the brighter surface of the fruiting body. a* and b* represent color components, with values ranging from [−60,60]. Among them, a* represents the displacement of mushrooms from green to red, where positive values represent the red color tone and negative values represent the green color tone. b* represents the displacement of the mushrooms from blue to yellow, where positive values represent the yellow color tone and negative values represent the blue color tone. The color parameters such as the hue angle (Hue), the color saturation (C), the color index (CCI) and the chromatism (△E) were calculated to further compare color differences of the mushrooms exposed to different light treatments. The calculation formula was as follows:

$$\mathrm{Hue}=\tan^{–1}\left(b^{*}/a^{*}\right),\;\mathrm{if}\;a^{*}>0;\;\mathrm{and}\;180+\tan^{–1}\left(b^{*}/a^{*}\right),\;\mathrm{if}\;a^{*}<0$$

$$C=\sqrt{a^{*2}+b^{*2}}$$

$$CCI=1000\;x\;a^{*}/\left(L^{*}\;x\;b^{*}\right)$$

$$\Delta E=\sqrt{\Delta L^{*2}+\Delta a^{*2}+\Delta b^{*2}}$$

### 2.3 Determination of nutrient content and extracellular enzyme activities

0.1 g mushroom tissue (ground in liquid nitrogen) mixed with 0.9 mL PBS buffer (pH = 7.4) were centrifuged at 4°C and 8,000 rpm/min for 30 min, then the supernatant was collected and stored at 4°C for use. The nutritional indicators and extracellular enzyme activities of *L. decastes* were determined using the Elisa assay kit purchased from Shanghai C-reactive Biotechnology Co. Ltd. The contents of crude polysaccharides (19), crude proteins (19), and total triterpenes (20) were determined according to the instructions of the biochemical analysis kit. The activities of extracellular enzymes including cellulase, hemicellulase, laccase, manganese peroxidase, lignin peroxidase and amylase were measured according to the instructions of the enzyme-linked immunosorbent assay kit (21–25).

### 2.4 Photoreceptor related gene expression analysis by quantitative real-time reverse-transcription polymerase chain reaction (qRT-PCR)

The total RNA of *L. decastes* was extracted using a polyphenolic polysaccharide plant total RNA extraction kit (Magen Biotechnology Co., Ltd., Guangzhou, China). The real-time PCR primers were designed using Premier 6.0, and gene quantitative analysis was performed on a fluorescence quantitative PCR instrument (Applied Biosystems, USA) using the SYBR green method. The 10 μL reaction mix was obtained using 5 μL of 1 × SYBR Green Master Mix, 1 μL of upstream and downstream primers, 2 μL of cDNA, and RNase-free Water. The relative transcript levels were calculated using the 2^−△△^CT method (26). GPD gene was used as internal gene in the test. The design of primer sequences for target gene and internal parameter gene was shown in Supplementary Table S1.

### 2.5 Statistical analysis

The relative spectral curve was extracted using Avasoft 8, and data was organized and plotted using Excel and SPSS Statistics 22. Cluster analysis was performed using Hiplot, and correlation analysis was performed using Origin 2021. The data was presented as mean ± errors.

## 3 Results

### 3.1 The growth and development of *Lyophyllum decastes* under different light treatments

**Figure 1:**
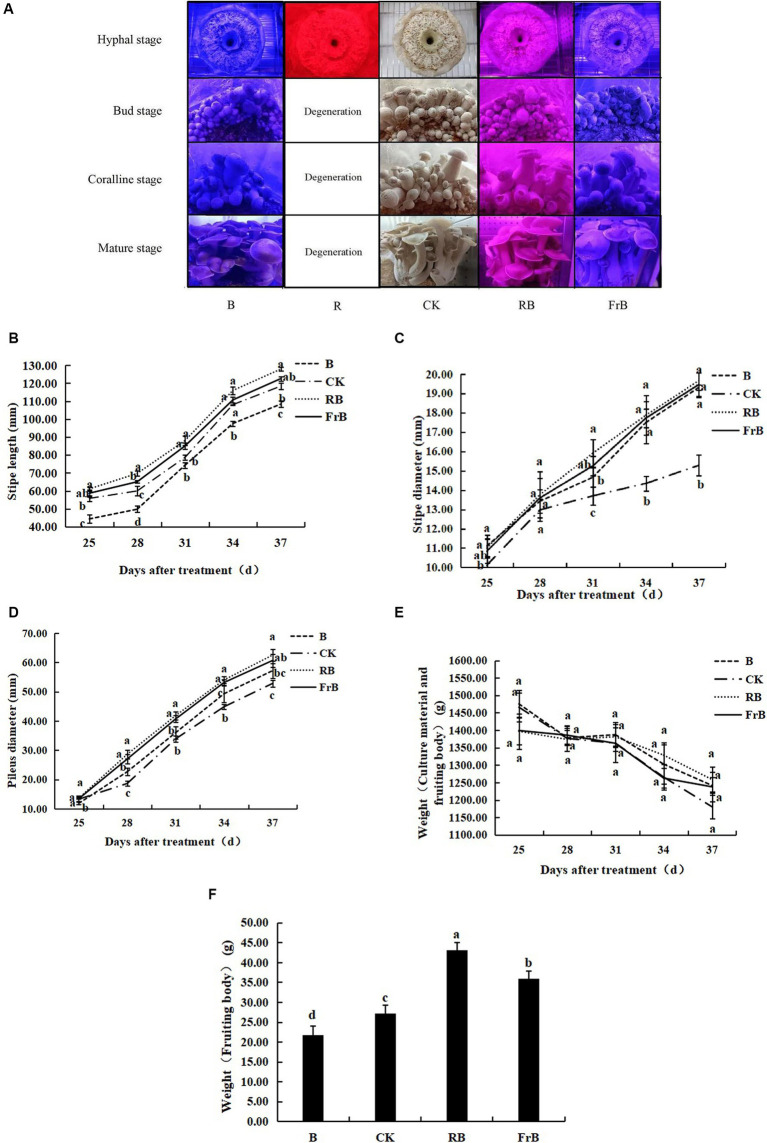
Growth and development of *L. decastes* under different light qualities. **(A)** Photos of *L. decastes* at different stages; **(B)** The stipe length of *L. decastes* at 25, 28, 31, 34, and 37 days after treatment (DAT); **(C)** The stipe diameter of *L. decastes* at 25, 28, 31, 34, and 37 DAT; **(D)** The pileus diameter of *L. decastes* at 25, 28, 31, 34, and 37 DAT; **(E)** The weight of culture material and fruiting body of *L. decastes* at 25, 28, 31, 34, and 37 DAT; **(F)** The weight of fruiting body of *L. decastes* at 37 DAT. Different lowercase letters indicate significant differences between groups (*p* < 0.05). The following figure is the same.

As shown in Figure 1A, the mycelium of mushroom treated with monochromatic red light (R) degenerated and the activity of fungal skin decreased. The primordium formation was inhibited and no fruiting body formed under R treatment, indicating that monochromatic red light was not conducive to the growth of *L. decastes*.

As shown in Figure 1B, the stipe length was increased by RB and FrB, while decreased by B, compared with the control. The stipe length of mushroom exposed to RB treatment kept the longest throughout the entire growth period, while that subjected to B treatment displayed the lowest. At 37 DAT, the stipe length of mushroom under RB and FrB treatments was increased by, respectively, 8.0 and 3.5% relative to the control, while that under B was significantly decreased by 8.5% (*p* < 0.05). It indicated that monochromatic blue light combined with red light or far-red light promoted the elongation of the stipe, on the contrary, the monochromatic blue light restrained the stipe elongation. The highest elongation rate of stipe was detected during the mature stage which was 5.7–6.6 mm·d^−1^ under different treatments.

The diameter of both stipe and pileus were increased by all the treatments compared with the control, displaying an order as RB > FrB > B > CK. At 37 DAT, the stipe diameter of mushroom exposed to RB, B and FrB treatments was significantly increased by, respectively, 28.7, 27.4 and 26.4% compared with the control (*p* < 0.05). The highest growth rate of stipe diameter was detected during the bud stage which was 0.9–1.0 mm·d^−1^ under different treatments (Figure 1C). At 37 DAT, the pileus diameter of mushroom exposed to RB, B and FrB treatments was significantly increased by, respectively, 18.3, 14.8 and 8.2% compared with the control (*p* < 0.05). The highest growth rate of pileus diameter was observed during the coralline stage which was 3.4–4.7 mm·d^−1^ under different treatments (Figure 1D).

The total weight of nutrients and fruiting bodies declined with the nutrient consumption in the bacterial bag over time, however, no significant difference was detected among different treatments (Figure 1E). In addition, as regards of the body weight at harvest, the weight of fruiting bodies subjected to RB and FrB treatments were significantly increased by, respectively, 58.2 and 31.9% (*p* < 0.05) compared to the control. On the contrary, B treatment significantly decreased the fruiting body weight by 20.2% relative to the control (*p* < 0.05) (Figure 1F). On the whole, compared with white light, blue light mixed with red light or far-red light were beneficial for increasing the size and weight of *L. decastes*. Monochromatic blue light inhibited the stipe elongation and the weight accumulation of the fruiting body, but promoted the lateral growth of *L. decastes* compared with white light.

### 3.2 The coloring of pileus and stipe of *Lyophyllum decastes* under different light treatments

Color is an important factor affecting the appearance and commercial value of mushrooms. C represents color saturation, with higher values indicating higher chromaticity and lower values indicating a color closer to gray. Hue represents the color angle, reflecting the coloring of the fruiting body. CCI stands for color index which can be used to evaluate the color changes of mushroom, and the color ratio a*/b* is the comprehensive color index. ∆E represents color difference, the larger the value, the greater the color difference. As shown in Table 2, all color parameters showed higher in the pileus than the stipe, which might be due to the stipe being obscured by the pileus and receiving less light. Compared with the control, mushroom pileus exposed to B and FrB treatments showed significantly higher value of Hue, CCI, a^*^/b^*^ and ∆E, together with darker simulated color (*p* < 0.05). It indicated that monochromatic blue or mixed blue and far-red light irradiation was beneficial for the coloring of the pileus. In contrast, the color change of stipe under different treatments was not as significant as that of the pileus from the result of simulated color.

**Table 2:** The color and simulated color of the pileus and stipe of *L. decastes* under different light qualities.

	Treatment	C	Hue	CCI	a^*^/b^*^	∆E	Simulated color
Pileus	B	21.0 ± 0.68a	3.53 ± 2.00b	5.86 ± 0.86a	0.30 ± 0.03a	16.84 ± 2.78a	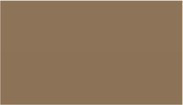
	CK	21.39 ± 1.18a	0.32 ± 0.10c	3.19 ± 0.29b	0.22 ± 0.02b	0d	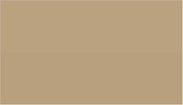
	RB	21.73 ± 0.54a	0.65 ± 0.73c	3.66 ± 0.48b	0.23 ± 0.03b	4.16 ± 1.13c	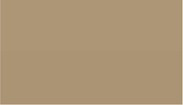
	FrB	22.40 ± 1.14a	8.30 ± 1.15a	5.96 ± 0.06a	0.33 ± 0.00a	12.45 ± 1.87b	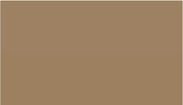
Stipe	B	16.86 ± 1.21a	1.70 ± 0.80a	0.84 ± 0.19a	0.06 ± 0.02a	3.66 ± 2.37ab	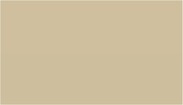
	CK	19.96 ± 1.14a	0.15 ± 0.11b	0.99 ± 0.33a	0.08 ± 0.02a	0b	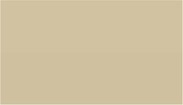
	RB	17.90 ± 2.61a	0.12 ± 0.07b	0.69 ± 0.42a	0.06 ± 0.03a	4.13 ± 3.13ab	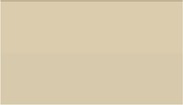
	FrB	16.76 ± 1.12a	0.30 ± 0.17b	1.02 ± 0.17a	0.08 ± 0.01a	7.45 ± 2.19a	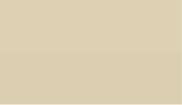

Different letters in the same industry indicate significant differences (*p* < 0.05), as shown in the table below. Calculate ∆E using CK as the standard.

### 3.3 The reflectance of pileus and stipe of *Lyophyllum decastes* under different light treatments

**Figure 2:**
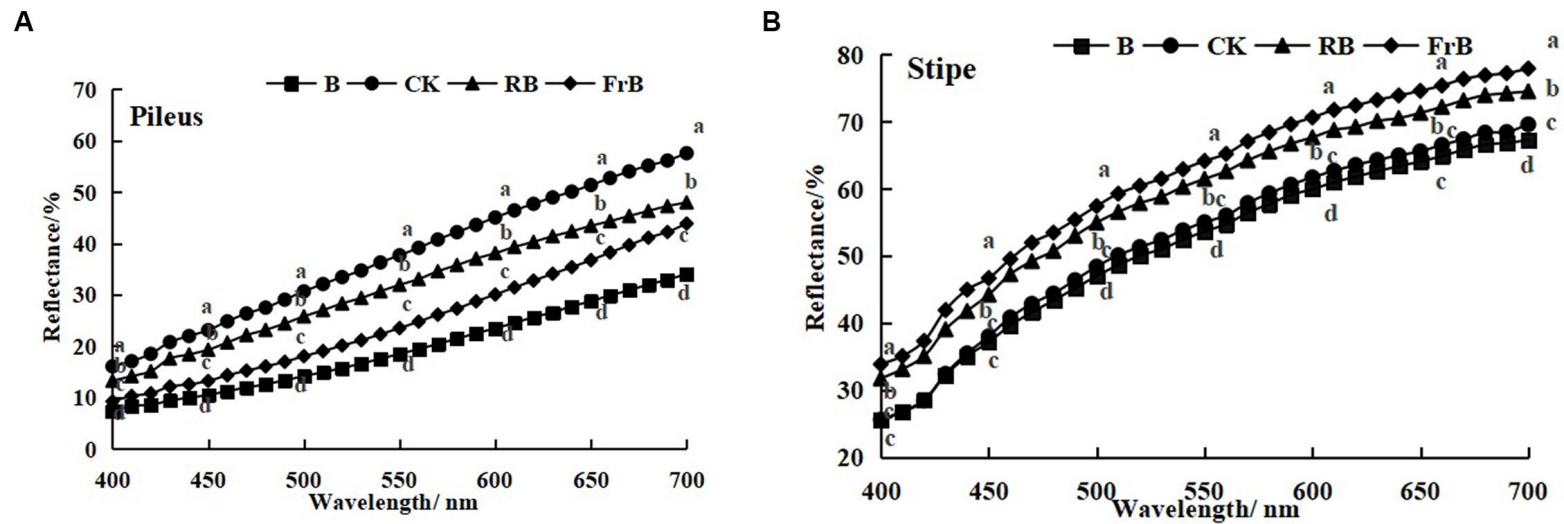
Reflection spectra of the pileus **(A)** and stipe **(B)** of *L. decastes* under different light qualities.

As shown in Figure 2, the reflection spectrum curves of mushroom were basically similar among treatments, and the reflectance of the pileus and stipe increased with the increasing wavelength. The reflectance of mushroom pileus in each treatment was lower than that of the control, as follows: CK > RB > FrB > B, the difference between each treatment reached a significant level (*p* < 0.05). This indicated that light treatments especially B and FrB enhanced the light absorption of pileus (Figure 2A), which was corresponded with the color parameters in Table 2. Similarly, the reflectance of the stipe was also found the lowest in mushroom exposed to B (significantly lower than the other treatments, *p* < 0.05), it might imply that monochromatic blue light was conducive for light absorption of mushroom. The reflectance difference in the stipe among treatments seemed smaller than that of the pileus, indicating that the pileus was more sensitive to light quality than the stipe (Figure 2B).

### 3.4 The nutrient quality of *Lyophyllum decastes* under different light treatments

**Figure 3:**
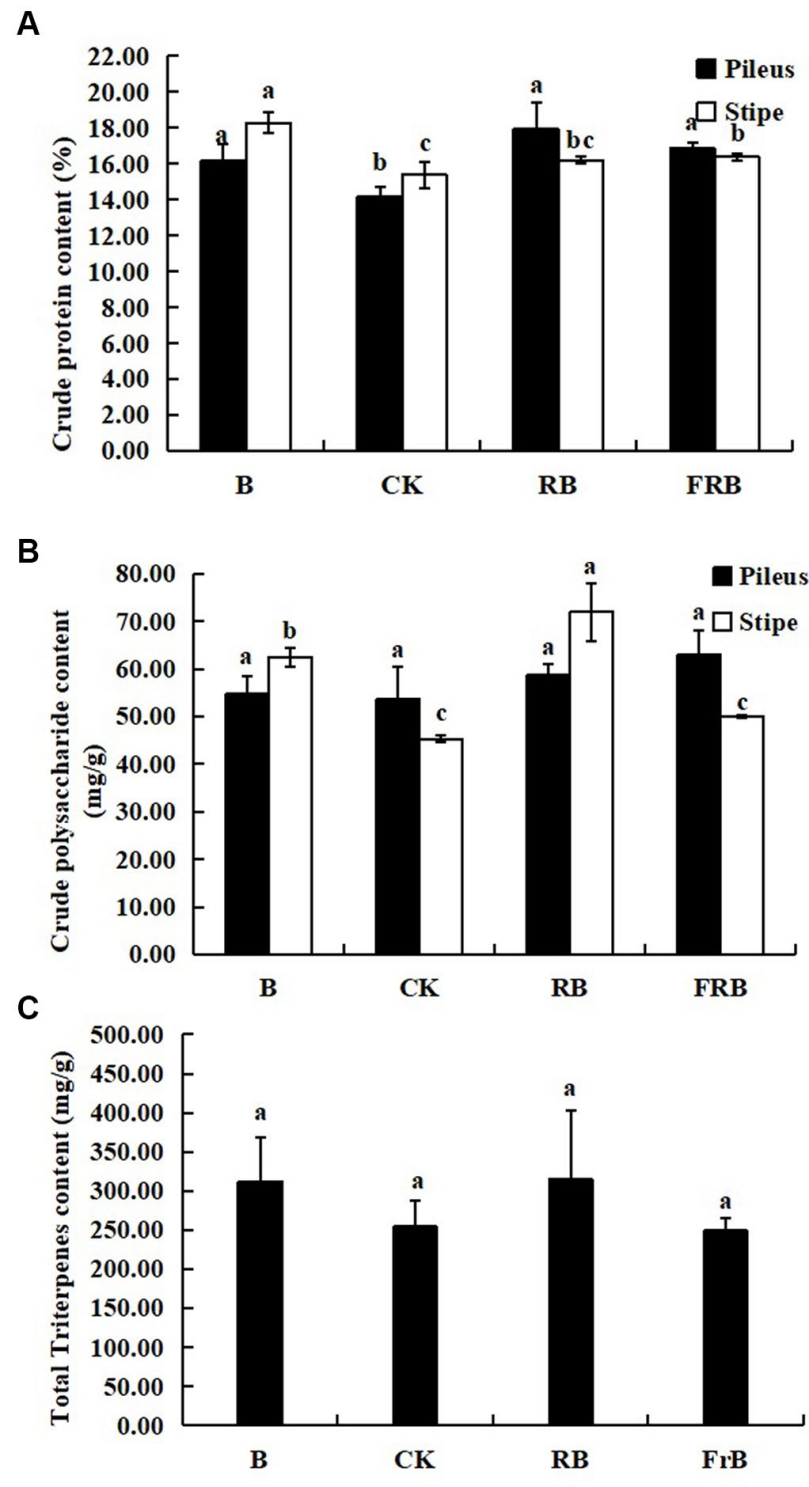
The contents of crude protein **(A)**, crude polysaccharide **(B)**, and total triterpenoid **(C)** in *L. decastes* under different light treatments.

As seen in Figure 3, all treatments raised the contents of crude protein, crude polysaccharide and total triterpenoids in the fruiting bodies in various degrees compared with the control. The crude protein content in pileus exposed to B, RB and FrB treatments was significantly increased by, respectively, 14.0, 26.5 and 18.8% (*p* < 0.05) in relative to the control, while that in stipe was increased by, respectively, 19.0, 5.3 and 6.5% (Figure 3A).

The crude polysaccharide content in pileus exposed to B, RB and FrB treatments was increased by, respectively, 2.1, 9.4 and 17.4% compared with the control, however no significant difference was observed. B and RB treatments significantly enhanced the crude polysaccharide content in stipe by, respectively, 38.0 and 58.8% (*p* < 0.05) compared with the control (Figure 3B). In addition, no significant difference was detected in the content of total triterpenoids in the fruiting bodies subjected to different light treatments (Figure 3C).

### 3.5 The extracellular enzyme activities of *Lyophyllum decastes* under different light treatments

**Figure 4:**
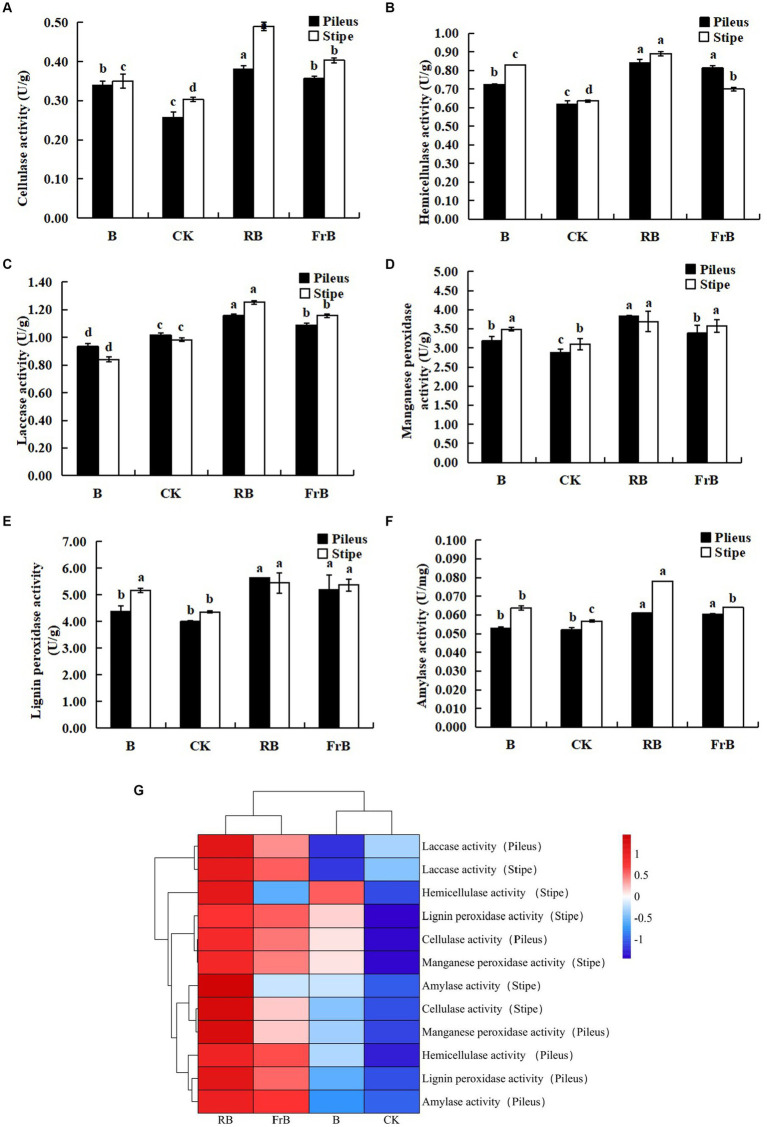
The enzyme activities of cellulase **(A)**, hemicellulase **(B)**, laccase **(C)**, manganese peroxidase **(D)**, lignin peroxidase **(E)** and laccase **(F)** in *L. decastes* under different light treatments, and the cluster heatmap of extracellular enzyme activity **(G)**.

As shown in Figure 4, the activities of cellulase, hemicellulase, laccase, manganese peroxidase, lignin peroxidase and amylase were all significantly (*p* < 0.05) raised in fruiting bodies exposed to RB and FrB treatments compared with the control, and the highest activities for these six extracellular enzymes were all detected under RB treatment, which were, respectively, 13.7% ~ 46.2% (in pileus) and 19.0% ~ 63.3% (in stipe) higher than those treated with the control. The increase of extracellular enzyme activities stimulated the degradation of the culture medium, thereby promoting the growth of fruiting bodies, which might explain the significantly higher weight of fruiting bodies in RB and FrB treatments. In contrast, the laccase activity of the fruiting body subjected to B treatment was significantly decreased compared with the other treatments, it might indicate that short wavelength irradiation was not conducive to the enhancement of laccase activity.

### 3.6 The relative expression level of photoreceptor genes in *Lyophyllum decastes* exposed to different light treatments

**Figure 5:**
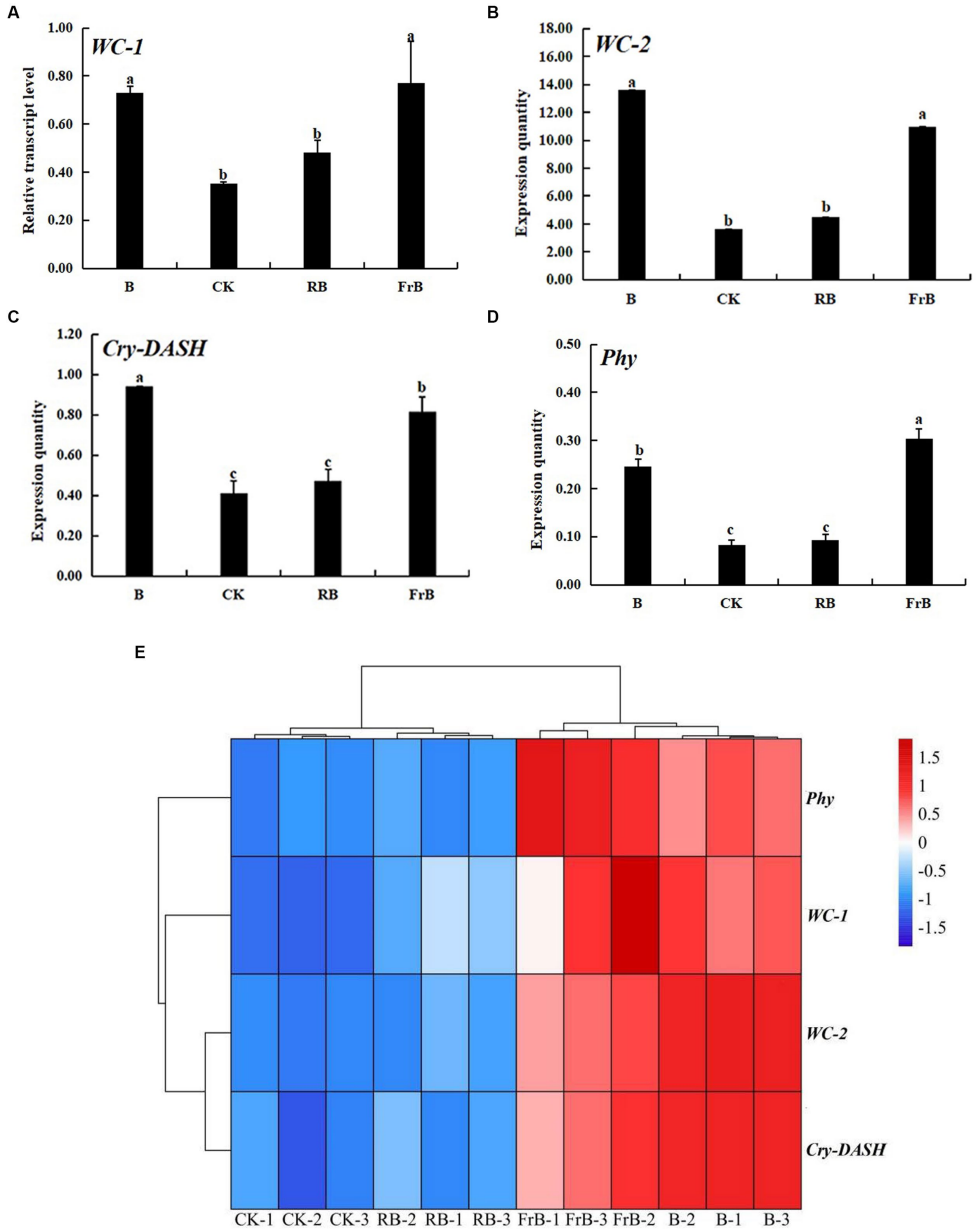
The relative expression levels of photoreceptor genes: *WC-1* **(A)**, *WC-2* **(B)**, *Cry-DASH* **(C)** and *Phy* **(D)** in *L. decastes* under different light treatments, and the cluster heatmap of photoreceptor genes **(E)**.

To further investigate the responses of *L. decastes* to different light qualities, RT-PCR technology was used to analyze the expression of photoreceptor genes in the fruiting bodies under different light qualities. As shown in Figure 5, B and FrB treatments significantly increased the expression levels of both blue light photoreceptor gene (*WC-1, WC-2 and Cry-DASH*) and red light photoreceptor gene (*Phy*) compared with the control (*p* < 0.05). The highest expression levels of *WC-2* and *Cry-DASH* were both detected in mushroom exposed to B treatment, significantly increased by 3 times and 1 times compared to the control (*p* < 0.05), respectively. The highest expression level of *WC-1* and *Phy* were both detected in mushroom exposed to FrB treatment, significantly increased by 1 time and 3 times relative to the control (*p* < 0.05), respectively. In contrast, no significant difference was detected in the expression level of photoreceptor genes in mushroom exposed to RB treatment compared to the control. The better coloring and higher light absorption of pileus subjected to B and FrB observed in Table 2 and Figure 2 might be due to the up-regulated expression level of photoreceptor genes by the two treatments.

### 3.7 Correlation analysis among the phenotype, nutrient qualities and extracellular enzyme activities of *Lyophyllum decastes* exposed to different light treatments

**Figure 6:**
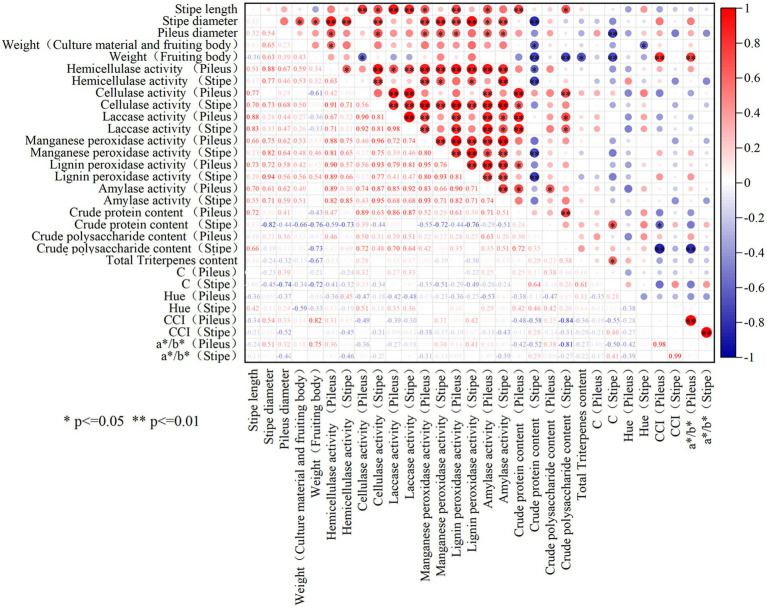
The correlation analysis among the phenotype, nutrient qualities and extracellular enzyme activities of *L. decastes*. * represents *p*-value less than 0.05, **represents *p*-value less than 0.01.

As shown in Figure 6, significant positive relationship was observed between the stipe length and the activities of laccase and cellulase in the stipe (*p* < 0.05). The diameter of the stipe and pileus was significantly positively correlated with the activities of cellulase, hemicellulase, manganese peroxidase, lignin peroxidase as well as amylase (*p* < 0.05). Moreover, the weight of the fruiting body was positively correlated with the activities of hemicellulase, manganese peroxidase, and lignin peroxidase in the stipe and pileus. It can be seen that the size and weight of *L. decastes* seemed generally positively correlated with extracellular enzyme activities.

As for the correlation between the nutrients and extracellular enzyme activity in the fruiting body, it was observed that the crude protein content was significantly positively correlated with the activities of cellulase and laccase in pileus, while the crude polysaccharide content was significantly positively correlated with the activity of amylase in pileus (*p* < 0.05). However, the crude protein content was negatively correlated with the activities of hemicellulase, manganese peroxidase, or lignin peroxidase in the stipe. Moreover, there is no significant correlation between triterpenoid substances and extracellular enzyme activity in the fruiting body. Therefore, the interaction mechanism between extracellular enzymes and the nutrient accumulation in the fruiting body was relatively complex.

## 4 Discussion

The fruiting stage of edible fungi is light sensitive stage, light can either stimulate or inhibit fungal development, so light is an important factor in the growth and development of edible fungi. Our study indicated that the highest average growth rate of stipe diameter, pileus diameter, and stipe length of *L. decastes* was, respectively, detected at bud stage (DAT 15-Day 25), coralline stage (Day 26–Day 31) and the mature stage (Day 32–Day 37) regardless of different treatments. Arjona et al. (27) reported that blue light was the triggering signal for the formation of *Pleurotus ostreatus* primordia, and the formation of primordia could not be achieved in a light environment lacking blue light. Ellis et al. (28) found that blue light with wavelengths of 440–470 nm was the most favorable for the formation of *Coprinus comatus* primordia. Kim et al. (29) found that blue light irradiation increased the expression level of FAD-NAD binding proteins related to primordial formation in *Lentinula edodes* by four times compared with the darkness. Li et al. (30) also observed that blue light up-regulated the expression of conidiation and hydrophobin proteins, which was related to the composition of fungal cell wall and cell membrane synthesis during the primordial formation in *Trichoderma*. These studies indicated that blue light played an important role in the formation of the primordium of edible fungi. In the present study, normal primordia formation of *L. decastes* occurred under CK, B, RB and FrB treatments. However, the mycelium of *L. decastes* degenerated and the fungal skin activity decreased under R treatment. It indicated that blue light was indispensable during the primordium formation of *L. decastes*, confirming the importance of blue light for edible fungi mentioned above. In addition, studies also have shown that several important biosynthetic pathways in mushrooms such as the membrane transport protein synthesis and the amino acid biosynthetic were found inactive under monochromatic red light irradiation. The function of membrane transporters was to perceive external stimuli and transmit signals to cells, maintaining the activity of mycelial (31, 32). Therefore, the degradation of *L. decastes* mycelium under R treatment in this study might be due to the lack of blue light irradiation or a decreased expression level of membrane transporter protein in *L. decastes* caused by monochromatic red light.

Our study found that RB treatment significantly enhanced the size and weight of the *L. decastes* fruiting body compared with the control (*p* < 0.05). On the contrary, B treatment significantly decreased the stipe length and the fruiting body weight. This was consistent with Jang et al.’s study (33), which showed that the stipe of *Hypsizygus marmoreus* was the shortest under the pure blue light compared with the other light treatments such as mixed blue and white light, mixed green and white light, combined blue and green light. It implied that although blue light acted a crucial role in the primordium formation of *L. decastes*, monochromatic blue light was not optimum light quality for the size or weight of fruiting body of *L. decastes*. In addition, the correlation analysis showed that the stipe length, the diameter of stipe and pileus were positively correlated with the activities of cellulase, hemicellulase, laccase, manganese peroxidase, lignin peroxidase, and amylase. Meanwhile, the fruiting body weight was positively correlated with the activities of hemicellulase, manganese peroxidase, and lignin peroxidase in both pileus and stipe. In the resent study, the highest extracellular enzyme activity accompanied with the highest size and fruiting body weight were all detected in *L. decastes* treated with RB treatment. The mixed red and blue light might promote the degradation of the cultivation materials and absorption of nutrients by *L. decastes* via increasing the activities of the extracellular enzyme, which might account for the best growth and weight of the fruiting bodies detected under RB treatment.

The color of edible mushroom is one of the main factors affecting consumer choices (34). Carotenoid is one of the main pigments that contribute to the coloration of fungi, providing characteristic yellow, orange or reddish colors (35). Studies have shown that the synthesis of carotenoids in *Fusarium* and *Cordyceps militaris* was associated with the blue light signaling pathway and was synergically regulated by blue light photoreceptor, while red light wave band was ineffective for the carotenoids synthesis (36, 37). The current study displayed that higher color spectral parameters such as Hue, CCI, a */b *, and ∆ E values as well as deeper simulated color of the pileus were detected in *L. decastes* under B and FrB treatments compared with the other treatments. It demonstrated that blue light or mixed far-red and blue light were beneficial for the coloring of *L. decastes*. This might be due to that B or FrB irradiation up-regulated the expression level of light photoreceptor genes such as *WC-1*, *WC-2* and *Cry-DASH,* which might participate to regulate carotenoid biosynthesis pathway in edible fungi (38).

Light quality not only affected the morphology formation of edible fungi, but also acted on the synthesis and accumulation of nutrient substance in the fruiting body. Tang et al. (39) used transcriptomics to study the photoresponse mechanism of *Lentinus edodes* and found that light would affect the transportation and metabolism of carbohydrates. The studies by Wang et al. (40) and Zhu et al. (41) showed that blue light was conducive to the synthesis of polysaccharides and protein in *Ganoderma lucidum* and *Hypsizygus marmoreus* compared with red light. Similarly, our study found that the contents of crude protein and crude polysaccharide in *L. decastes* exposed to B, RB, FrB treatments were all increased compared with the control. The possible reason was that organic metabolism related genes such as hydrophobin genes (*SC1* and *SC3*), lignin-modifying genes (*LAC1*, *LCC2* and *LCC3*) and Tyrosinase-encoding genes (*TYR1* and *MELC2*) were up-regulated under the those light qualities (29, 39, 42, 43).

Extracellular enzyme activity reflects the ability of mushrooms to absorb and utilize small molecule nutrients. It has been reported that extracellular enzyme stimulated the degradation of the culture medium, thereby promoting the organic metabolism and the growth of fruiting bodies (14). Cao et al. (44) found that higher activities of cellulase and laccase appeared with higher contents of polysaccharides in *Lentinula edodes*. In the present study, the correlation analysis in Figure 6 showed that the crude protein content was significantly positively correlated with the cellulase and laccase activities in the pileus, while the crude polysaccharide content was significantly positively correlated with the amylase activity in the stipe (*p* < 0.05). The results confirmed the positive correlations between the extracellular enzyme and the organic metabolism. In addition, our study found that the activities of cellulase, hemicellulase, manganese peroxidase, lignin peroxidase and amylase were all raised in fruiting bodies exposed to B, RB and FrB compared with the control. Thus, the increased activity of cellulase, hemicellulase, peroxidase and amylase in *L. decastes* treated with B, RB, FrB may also accounted for the higher contents of organic substance observed in those light treatments. Xie et al. (45) studied the effects of blue light on the activity of manganese peroxidase in *Pleurotus eryngii*, to find that blue light inhibited the activity of manganese peroxidase compared with darkness. Ramírez et al. (46) reported that blue light significantly reduced the activity of lignin peroxidase in *Phanerochaete chrysosporium Burds* in relative to white light. On the contrary, Gan et al. (47) and Zhu et al. (48) reported that blue light increased the activity of amylase in *Pseudopestalotiopsis theae, Fusarium solani, Xylaria venustula* and *Aspergillus niger* compared with the white light. The results in our study displayed that the activities of the manganese peroxidase, lignin peroxidase and amylase were all increased in *L. decastes* treated with B compared with the control. This might indicate that the effects of light quality on extracellular enzymes in edible fungi was variety dependent. Furtherly, when comparing the effects of these three light qualities (B, RB and FrB) on extracellular enzymes in the current study, it was found that the up-regulation effect of RB and FrB were more significant compared with B. It implied that blue light mixed with red light or far-red light were more effective to the extracellular enzyme activities in *L. decastes*.

Light signals were first transmitted into cells through photoreceptor proteins, and then the protein complexes acted as transcription factors to regulate the expression of many downstream genes, thereby regulating various life activities such as the development and nutrient synthesis in edible fungi (49–51). The results in our study showed that all treatments up-regulated the expression levels of the four photoreceptor genes (*WC-1*, *WC-2*, *Cry-DASH*, and *Phy*) compared with the control. Additionally, it was noticed that all treatments enhanced the activities of extracellular enzymes such as cellulase, hemicellulase, manganese peroxidase, lignin peroxidase and amylase in *L. decastes.* This may because that photoreceptor genes were involved in the regulation of extracellular enzyme (52). Nevertheless, since light environment elements include light intensity, light period, light quality and light distribution, the current study only analyzed the effects of light quality on *L. decastes*, further studies are needed to reveal the effects and mechanisms of the other light factors on the growth and development of *L. decastes.*

## 5 Conclusion

R led to the degeneration of the mycelium and decreased the activity of fungal skin, without forming the primordia. RB significantly promoted the increase of volume and weight in *L. decastes* and up-regulated the activities of extracellular enzyme in mushrooms, while B significantly decreased the stipe length and the weight of mushroom fruiting body compared to the white light. B or FrB were beneficial for the coloring of *L. decastes*, in that blue light photoreceptors (*WC-1, WC-2* and *Cry-DASH*) which synergically regulated the main pigments contributing to the coloration of fungi were up-regulated by B and FrB. On the whole, the largest volume and weight as well as the highest contents of crude polysaccharide, crude protein and total triterpenoids were all detected in the *L. decastes* fruiting bodies treated with RB, compared with the other treatments.

## Data availability statement

The original contributions presented in the study are included in the article/Supplementary material, further inquiries can be directed to the corresponding authors.

## Author contributions

XC: Writing – original draft, Writing – review & editing. YL: Writing – original draft, Writing – review & editing. WG: Data curation, Software, Writing – review & editing. MW: Data curation, Software, Writing – review & editing. JZ: Data curation, Software, Writing – review & editing. XZ: Supervision, Writing – review & editing. WZ: Supervision, Writing – review & editing.
