# Supplementary material for: The development and nutritional quality of Lyophyllum decastes affected by monochromatic or mixed light provided by light-emitting diode
Source: Front Nutr. 2024 May 27;11:1404138. doi: 10.3389/fnut.2024.1404138 (PMC11163063; doi:10.3389/fnut.2024.1404138)
Supplement: Supplementary file 1 [file Data_Sheet_1.docx]

*Supplementary Material*


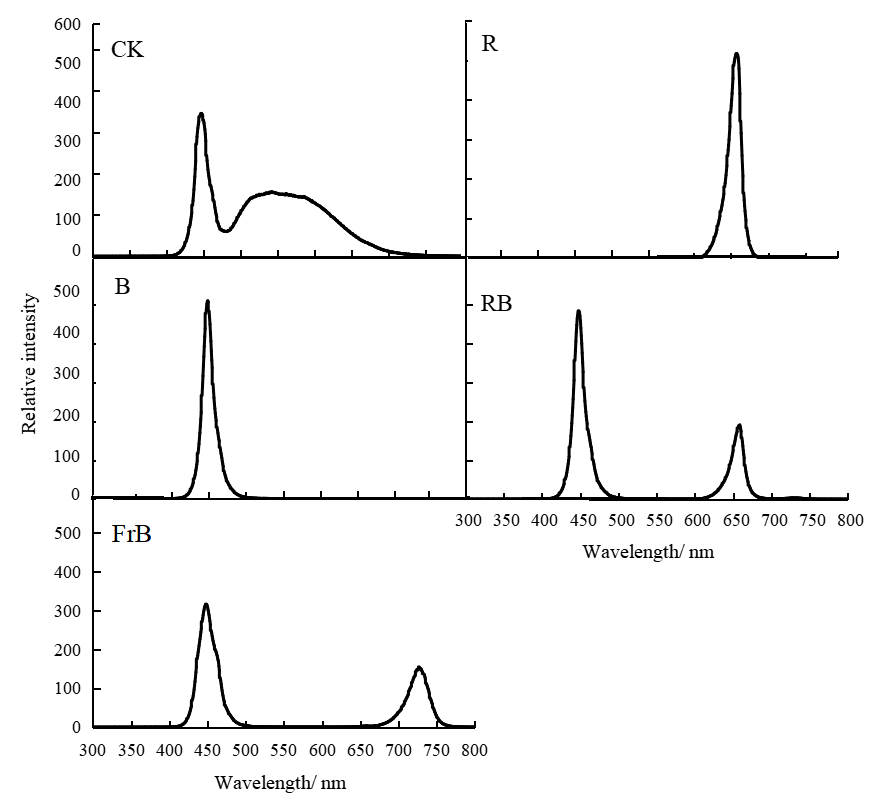
Figure S1 LED spectral distribution map for each treatment.

Table S1 Specific primers for real-time quantitative PCR

| **Primer** | **Sequence（**5'-3'**）** |
| --- | --- |
| GPD-F | CCATCCACGCTTCCACTG |
| GPD-R | TAGACGGGACCGAAACAC |
| FfWC-1-F | TACCCAAGAAACCTCCGCCTATCC |
| FfWC-1-R | TGAGCGAGACAACGAGGAAGAAATC |
| FfWC-2-F | CTACCCTCCCTCGTTGCCTCTG |
| FfWC-2-R | CGTCCTCATTGCGTTCCGTCAG |
| FfCry DASH-F | GAACTTCACGCACCTCATCC |
| FfCry DASH-R | ACCGCATCGCCAGAACTT |
| Ff phy-F | CCTCCTCGGTATCTCTTCTCGCTAG |
| Ff phy-R | TTGAAATCCTGGTCGCTGTCTGATG |
